# Correlations between Malocclusion and Postural Anomalies in Children with Mixed Dentition

**DOI:** 10.3390/jfmk4030045

**Published:** 2019-07-19

**Authors:** Sergio Sambataro, Salvatore Bocchieri, Gabriele Cervino, Rosario La Bruna, Alessandra Cicciù, Marcella Innorta, Benedetto Torrisi, Marco Cicciù

**Affiliations:** 1 Department of Biomedical and Dental Sciences and Morphological and Functional Imaging, Messina University, 98100 Messina ME, Italy; 2 Private Practice, 89100 Reggio Calabria RC, Italy; 3 Bronte UOC Orthopedic Hospital, 95034 Bronte CT, Italy; 4 Business and Economics Department, Statistics Economic Area-Catania University, 95100 Catania CT, Italy

**Keywords:** malocclusion, mixed dentition, scoliosis, posture

## Abstract

The aim of this study was to investigate the possible relationship between malocclusion and body posture anomalies. The original sample involved 127 children (45 males and 82 females) with mixed dentition. Clinical examination of oral cavity was performed by an orthodontist, who recorded molar and canine relationship, cross-bite, lower middle-line deviation, and centric relation (CR) considering mono or bilateral contacts in CR. Orthopedic examination of the body posture was clinically carried out by an orthopedist who detected anomalies such as scoliosis, false scoliosis or paramorphism, kyphosis and lordosis. Of the 127 subjects of the sample, 18 children were orthopedically normal, 80 patients had false scoliosis, 22 scoliosis and 7 showed kyphosis. In our study, we don’t consider the 7 patients with kyphosis for the exiguity of the sample; so, our analysis was performed on 120 children (42 males and 78 females). The results obtained revealed that the cross-bite was more frequent when scoliosis became worse. We also found that the relationship between left cross-bite and contralateral side of deviation of the curve of the spine in subjects with scoliosis is statistically significant (*p* = 0.002). Furthermore, the relationship between lower midline and contralateral side of deviation of the curve of the spine in patients with false scoliosis is statistically significant (*p* = 0.003). In conclusion, it seems that posture anomalies are correlated to cross-bite and mandible abnormal position.

## 1. Introduction

Malocclusion and incorrect body posture are two very common issues in growing subjects and especially in patients with mixed dentition, where it is still possible to intervene to modify and correct both conditions. In order to perform a correct diagnosis and an orthodontic treatment plan, the relationships between occlusion and posture should be evaluated to establish the most appropriate strategy of treatment and an interdisciplinary approach between different healthcare professionals. Idiopathic scoliosis and secondary scoliosis had the highest frequency compared to all other spinal deformities [1,2]. Scoliosis is a three-dimensional disorder of the spine. The fulcrum of this pathology is the torsion of the vertebrae. Idiopathic scoliosis is a complex structural deformity of the spine that is modified on the three planes of space, and is defined as lateral curvature of the spine in the frontal plane greater than 10 degrees with vertebral rotation in the horizontal plane on a standing radiography. It mainly affects young subjects and is four times more frequent in females than in males. Females also have a 10 times higher risk of progression than males. Depending on the age of appearance it has been classified into three types: infantile (presenting from birth to 3 years), juvenile (presenting from 3 to 10 years) and adolescent (presenting from 10 years to skeletal maturity) [3,4].

The secondary form is due to diseases of connective tissue, neurologic and musculoskeletal disorders.

False scoliosis or paramorphism, is a different clinical condition in which the rotation is not present and it is caused by different lengths of the lower limbs, radiculopathy of spine, postural disorders, or inflammation [5].

The aim of this study is to search for possible correlations between malocclusions, which could lead to the onset of Temporomandibular Joint (TMJ) disorders [6,7,8,9,10,11,12,13,14] and incorrect body posture. If intercepted early, consolidation over time and surgical spine treatments can be avoided [15,16,17,18,19,20].

## 2. Materials and Methods

The clinical examination was carried out on 127 children (45 males and 82 females), in pre-pubertal stage, at the age of 9.8 years ± 8 months) in mixed dentition, with good general health (absence of craniofacial syndromes or other craniofacial anomalies, and we excluded patients with previous history of trauma to maxillo-facial district or to the spine). Each child underwent an orthopedic examination, followed by an orthodontic one.

Patients’ parents signed a consent for their child’s participation in the study, but an ethics committee’s opinion wasn’t required because only one additional clinical examination was done. Orthopedic screening was performed in an upright position, without clothes and without shoes, and was performed by an orthopedist who examined the young subjects. The asymmetry of the shoulders, the asymmetry of the hips, and obliquity of the pelvis with respect to overhanging, by evaluating the alignment of the line joining the shoulders, the line joining the scapulae and the line joining the iliac crests were recorded. The examination was completed with the analysis of the trunk in flexion, analyzing the possible presence of the hump and, therefore the site and the entity. At the end, the patient was observed in a lateral upright position for the evaluation of the physiological curves and their possible alterations (kyphosis and lordosis). In addition, the Bricot test was performed in order to evaluate the ligamentous hyper laxity of the fingers and of the hands and of the different lengths of the lower limbs.

The orthodontic data were recorded according to the Ricketts clinical examination, which takes into consideration numerous parameters; the ones that we took into account were:

1. The cross-bites, considered when posteriorly the top back teeth bite down inside the bottom back teeth. It occurs when the top teeth or jaw are narrower than the bottom teeth and can happen on one or both sides of the mouth [21].
2. The relationship between the upper dental arch and the lower dental arch according to Angle and defined as follows. Class I occurs when the mesiobuccal cusp of the upper first molar occludes with the buccal groove of the lower first molar. Class II occurs when the mesiobuccal cusp of the upper first molar occludes anterior to the buccal groove of the lower first molar. Class III occurs when the mesiobuccal cusp of the upper first molar occludes posterior to the buccal groove of the lower first molar [22].
3. Evaluation of habits, such as thumb sucking, tongue thrust, mouth breathing, lower lip sucking and nail biting.
4. Examination of soft tissues in order to detect the symmetry of the face, the mouth disharmonies, the lip imbalances, the mentalis habits and the perioral contractions.
5. The clinical deviation of the dental upper and lower midline from the midsagittal plane of the face.
6. The occlusal contacts (mono or bilateral) in maximum intercuspidation of the teeth.

The analysis of any interrelationships between postural anomalies and malocclusions were carried out by tabulating the data on special two-entry tables, one side of which refered to postural anomalies (scoliosis, lordosis, kyphosis, transverse planes of the back and different lengths of lower limbs) and the other to the orthodontic parameters previously listed in a descriptive way. Furthermore, in order to find any correlation between the malocclusions and the postural anomalies, we performed statistical analysis of the data with SPSS software (SPSS^®^ Inc., Chicago, IL, USA) performing chi-squared test, and the Pearson’s *r* with *p*-value were calculated.

## 3. Results

At the orthopedic examination, numerous young patients presented various postural problems. From severe to mild and without any clinical objectivity, therefore in the description of the orthopedic anomalies, reference will be made to the entity of these after having subdivided the subjects of the sample into groups to which they belong.

### 3.1. Evaluation of Postural Abnormalities

#### 3.1.1. Group of Patients with Severe Postural Abnormalities

This group consisted of 18 patients (7 males 11 females). The presence of a dorsal hump in all these subjects was found during the clinical examination. It was therefore advised to perform an X-ray examination of the column in its entirety. In 11 patients, the scoliotic curve also extended to the lumbar spine. Four of them had different lengths of the lower limbs of about 1.5 cm, with convexity of the curve on the same side. In 7 patients an important dorsal scoliosis was present.

#### 3.1.2. Group of Patients with Intermediate Postural Abnormalities

87 patients (26 males and 61 females) were in the original sample, but we didn’t consider 7 patients with kyphosis for the exiguity of the sample. The remaining 80 patients (23 males and 57 females) were included in this group and presented false scoliosis or paramorphism.

The orthopedic alterations were distributed in the males as follows. Seven were suffering from a dorsal scoliosis, and in 2 patients with dorsal scoliosis there was a tendency of hyperkyphosis and pain during neck flexion, extension and left lateral flexion.

The anomalies that we found in the remaining 14 patients were as follows. A dorsal false scoliosis was found in 2 of the patients. Six patients had a tendency of hyperkyphosis (2 patients had concomitant pain during the right and left lateral flexion of the cervical spine). Two patients had a dorsolumbar kyphoscoliosis which caused different lengths of the lower limbs of 3 and 3.5 cm. In 2 patients, a dorsolumbar kyphoscoliosis with a different length of the lower limbs of 1 cm was detected, and a dorsolumbar scoliosis with postural attitude of permanent lateral flexion of the left cervical spine was found in 2 patients.

The orthopedic alterations were distributed in the 57 females as follows. Thirty-two had a dorsal scoliotic curve, and some other alterations were concomitant. In 3 patients, there was an objective asymmetry of the right sternocleidomastoid, another 3 had the tendency of hyperkyphosis with back pain during flexion of the cervical spine. Three patients complained of cervical pain during right lateral rotation and another 3 patients had a hyperkyphosis paramorphism. Eleven patients presented with dorsolumbar scoliosis, and in 8 of them there was also a dorsal hyperkyphosis.

Of the remaining 14 patients, 9 had a dorsal false scoliosis (in 4 patients concomitant ipsilateral different lengths of the lower limbs of about 1.5 cm was found). A permanent right lateral flexion of the cervical spine was present in 3 patients, and in the last 2 patients a different length of the lower limbs lower of 1.5 cm was detected.

#### 3.1.3. Absence of Any Clinical Objectivity

The remaining 22 patients (8 males 14 females) were classified as orthopedically healthy.

### 3.2. Relationship between Types of Column Anomaly and Orthodontic Parameters

In the group of subjects with severe postural abnormalities it was recorded that 50% had a cross-bite, 50% had a deviation of the lower midline and 0% had unilateral contacts in centric relation (RC). In the group of patients with intermediate postural abnormalities it was detected that 25% had a cross-bite, 25% had deviation of the lower midline and 11.4% had unilateral contacts in RC. At the end, in the group of subjects without any clinical objectivity it was found that 33% had a cross-bite, 25% had deviation of the lower midline and 0% had unilateral contacts in RC.

To examine the results more analytically regarding the type of spine anomaly, the sample was also divided according to the type of column anomaly recorded at the orthopedic examination. For each group, the frequency of the orthodontic parameters taken into consideration was presented.

Therefore, we considered the subjects (22 patients) without any clinical objectivity (normal), with false scoliosis (80 patients) and with structured scoliosis (18 patients). In our analysis, we didn’t consider 7 patients with kyphosis for the exiguity of the sample.

A descriptive analysis was carried out, Table 1 shows the percentages of posterior cross-bites recorded in subjects with structured scoliosis (50%), compared to subjects with false scoliosis (25%), and healthy (33%). The deviation of the lower midline in maximum intercuspidation is more frequent in subjects with scoliosis (50%), while unilateral contacts in centric relation are more frequent in subjects with false scoliosis (Table 2).

In all three subgroups about molar relationship (Table 3), we can observe a very low frequency of Class III and a slight prevalence of Class II. It is particularly interesting that in patients with false scoliosis and with structured scoliosis, a frequent discrepancy between the molar relationship of one side and the other occurs. This is contrary to what was observed in patients without any abnormality of the spine. Regarding the differences between one or more transversal planes, the most relevant result is represented by the finding of a high frequency of cross-bites in subjects with asymmetry of more than one transversal plane (Table 4).

From statistical analysis of the data, we found that the cross-bite was more frequent when scoliosis became worse (from normal subjects, to false scoliosis, to scoliosis), and the Pearson chi-square test was significant with a value of 0.003. No other statistically significant relationship was detected (Table 5). The correlation between false scoliosis, scoliosis and cross-bite is evident with a Pearson’s *r* of 0.279, and it is statistically significant with a *p*-value of 0.002 (Table 6). We also found that the relationship between left cross-bite and contralateral side of the deviation of the curve of the spine in subjects with scoliosis is statistically significant with a value of 0.632 and a *p*-value of 0.005. We didn’t find the same result for the right cross-bite, probably due to the small size of our sample of subjects examined (Table 7). Another interesting relationship exists between bilateral cross-bite and the presence of false scoliosis or scoliosis. An *r* value of −0.0192 with a *p*-value of 0.036 indicates a negative correlation and demonstrates that in the bilateral cross-bite there is a symmetric posture of the mandible without shifting.

Furthermore, deviations of the lower midline were more frequent in patients with asymmetry of the line joining the shoulders and in subjects with a deviation of the spine relative to the overhanging (Table 8). Moreover, a statistically significant correlation exists between lower midline deviation and false scoliosis, with a value of 0.376 and a *p*-value of 0.001. As we found for the cross-bite, the relationship between left deviation of lower midline and contralateral side of the deviation of the curve of the spine with false scoliosis is statistically significant with a value of 0.327 and a *p*-value of 0.003. The high frequency of cross-bites and lower midline deviation indicates that in the sample we analyzed, the cross-bite was caused by a bilateral contraction of the upper arch responsible for the shift of the mandible on the affected side, causing a functional cross-bite (Table 9).

The correlations show a close interdependence between the orthodontic transverse anomalies (cross-bite, lower midline deviation) and those of the spine. This further demonstrates how an alteration of the mandibular posture may influence the neuromuscular kinematic chain of the total body [23].

## 4. Discussion

Early screening to intercept scoliosis is desirable and prevents patients from longer and more complex treatments and spinal surgery [24,25]. The diagnosis of scoliosis is based on a clinical and radiographic examination: the clinical evaluation assumes prominent relevance in the early diagnosis of some forms, the radiographic examination can be performed after the clinical evaluation. The clinical signs for the diagnosis of scoliosis are the hump, misalignment, the obliquity of the pelvis, asymmetry of the triangles of size, asymmetrical scapular profiles, bulging and the insufficiency of the abdominal muscles [3]. False scoliosis or paramorphism should not be confused with scoliosis, it is considered a para-physiological framework with spontaneous evolution, completely independent of structured scoliosis, it is always correctable in the supine position or in the movement of the trunk forward and, contrary to scoliosis, it is not foreseen by the deformity of the vertebral bodies [4,26].

From the tables, depending on the difference in level of one or more transversal planes we do not take into consideration the correlations between the incidence of occlusal anomalies and the difference of more than one transverse plane, while the lower midline deviations are very frequent in subjects with differences of the line joining the cingulum-scapula-humeral.

In our study, we found significantly high correlations between cross-bites and lower midline deviation in the group of patients with structured scoliosis and with false scoliosis, respectively, suggesting that the anomalies of the rachis somehow correlate with malocclusions such as contraction of the upper arch, and with a mandibular deviation in the three planes of space. It is also well known that the posterior cross-bite occurs in the baby dentition or in the early mixed dentition and is rarely self-corrected. It could be hypothesized that this orthodontic condition has a descending role in the onset of postural pathology [21].

Previous investigation showed that left-right asymmetries are among the most common anomalies in patients with scoliosis [27], in fact this condition seems to be also evident in the maxillo-facial district of subjects with unilateral cross-bite, lower midline deviation and facial asymmetries [28].

In Accordance to the results of the present study, Lippold et al. also showed that the degree of midline deviation is associated with the unilateral cross-bite and that the severe type of scoliosis was characterized by significantly higher presence of midline deviation. On the other hand, we didn’t find a relationship between unilateral Class II malocclusion associated with false scoliosis or scoliosis [28]. It is interesting to underline that in our sample we found a compensatory curve of scoliosis in the opposite side of the cross-bite, as reported by Hirschfelder [29].

It is well known that a relationship exists between mandibular lateral deviation, unilateral cross-bite and TMJ dysfunction. Experimental studies in rabbits tried to explain the origin and the mechanisms that cause the onset of an asymmetric growth of the head due to a unilateral cross-bite [30,31]. This aspect is particularly important in growing patients to avoid condylar compression that could cause structural asymmetry of the face [19,32]. Other research has shown that a unilateral cross-bite is associated with an asymmetrical condylar position, with an asymmetrical mandibular opening pattern [28,33], and this appears to reduce mandibular condylar growth [9], causing a shortening of the mandibular ramus from the side of the cross-bite [11,33]. An early correction of the cross-bite is therefore desirable [8,10,34] because an asymmetric occlusion causes an alteration of the growth that is responsible of mandibular and facial asymmetry [6,7,9,12,13], and subsequently also an asymmetry of the spine. For this type of correlation, the role of the lower jaw was emphasized, with the aim of correct posture of the whole body.

It’s interesting to note that a scoliotic curve has been developed after induction of a unilateral cross-bite in rats and these changes were observed after one week of unilateral alteration and were corrected after the rebalancing of the occlusal plane [33,35].

From the results of this paper we can state that a good occlusion is very important to maintain the stability of the kinetic chain of the body. Particularly, as we already showed, the position of premolars plays an important role in the stability of occlusion [36], and early orthodontic treatment could prevent the development of complex malocclusions, especially cross-bites [34,37,38,39,40,41].

Achieving a good occlusion is easier in growing patients and is a preventive way to reduce longer and more complex treatments, surgical procedures and the need to assume drugs, for the benefit of patients [42,43,44,45,46,47,48,49,50].

## 5. Conclusions

It is therefore evident that there is a correlation between scoliosis and malocclusions on the transversal plane but not on the sagittal plane, and the presence of these types of malocclusion imposes a postural evaluation of the patient by the orthodontist.

A limitation of our work was that we treated patients orthodontically, but we didn’t reevaluate them with orthopedic examination after orthodontic treatment and vice versa.

Further research could help to reveal more details on the correlations between body posture and malocclusions from a pathogenic and clinical point of view in order to clarify whether the diseases occur concomitantly or if one is dependent on the other, if the therapy in one of the two districts influences the other, and in particular, if orthodontic treatment of patients, especially with cross-bite, can improve or correct the false scoliosis. In case of scoliosis, after having performed the radiographic examination, it could be assessed whether orthodontic treatment improves the prognosis.

Thus, all the asymmetries observed in patients with false scoliosis and scoliosis indicate the need for an early orthodontic and orthopedic examination.

However, further research is desirable to clarify whether there is a causal relationship between occlusal changes and body posture.

## Figures and Tables

**Table 1:** Relations between scoliosis and cross-bite in our sample.

Rachis		Cross-Bite			
		Bilateral	Right	Left	Total
Normal 22	8M	2M	2M		6/18
	14F	2F			33%
False scoliosis 80	R conv.				
	13M	3M	3F	3M	
	25F		2M		
	L conv.				
	10M	3M	2F	1M	20/80
	32F	3F			25%
Scoliosis 18	R conv.				
	6F		2F	3F	
	L conv.				
	7M		2M		9/18
	5F		2F		50%

**Table 2:** Relations between scoliosis and lower midline deviation and contacts on RC in our sample.

Rachis		Lower Midline Deviation			Contacts on RC		
		R	L	Total	Bilateral	R	L
Normal 22	8M			5	8M		
	14F		5F	22%	14F		
False scoliosis 80	R conv.			20			
	13M		6M	25%	13M		
	25F	5F	5F		21F	4F	
	L conv.						
	10M	2M			7M		
	32F		2F		23F	9F	
Scoliosis 18	R conv.			9			
	6F	3F	2F	50%	6F		
	L conv.						
	7M		2M		7M		
	5F	2F			5F		

**Table 3:** Relations between scoliosis and molar relationship (according to Angle’s classification) in our sample.

Rachis		Molar Relationship R			Molar Relationship L		
		I	II	III	I	II	III
Normal 22	8M		8M			8M	
	14F	8F	6F		8F	6F	
False scoliosis 80	R conv.						
	13M	4M	7M	2M	4M	7M	2M
	25F	14F	5F	6F	20F	5F	
	L conv.						
	10M	2M	8M		3M	7M	
	32F	11F	18F	3F	11F	18F	3F
Scoliosis 18	R conv.						
	6F	6F			2F	4F	
	L conv.						
	7M		7M		3M	4M	
	5F		5F		2F	3F	

**Table 4:** Relations between differences in transversal planes and cross-bite in our sample.

Rachis		Cross-Bite			
		Bilateral	Right	Left	
Symmetry 16	9M	2M	1M	1M	5
	7F				33%
Asymmetric scapula-humeral track 17	R conv.				
	2M				
	8F		2F		2
	L conv.				
	2M				
	5F				11%
Deflection from overhanging 13	R conv.				
	5F				
	L conv.				2
	8F			2F	14%
More than one transversal plan 34	R conv.				
	5M	2M			
	14F		7F		
	L conv.				
	5M		5M		16
	10F			2F	47%

**Table 5:** Pearson chi-square test of our sample.

		Normal (22)	False Scoliosis (80)	Scoliosis (18)	Total (120)	Pearson Chi-Square	*p*-Value
Bilateral Cross-bite	no	18	74	18	110	4.502	0.105
	yes	4	6	0	10		
Cross-bite	no	20	66	9	95	11.662	0.003
	yes	2	14	9	25		
Cross-bite R	no	20	70	12	102	5.740	0.057
	yes	2	10	6	18		
Cross-bite L	no	22	76	15	113	5.310	0.070
	yes	0	4	3	7		
Lower midline deviation	no	17	60	9	86	4.940	0.085
	yes	5	20	9	34		
Lower midline deviation R	no	17	73	13	103	5.999	0.050
	yes	5	7	5	17		
Lower midline deviation L	no	22	67	14	103	4.877	0.087
	yes	0	13	4	17		
Contacts on RC bilateral	no	0	33	0	33	22.759	0.000
	yes	22	47	18	87		
Contacts on RC Right	no	22	50	18	90	20.000	0.000
	yes	0	30	0	30		
Contacts on RC Left	no	22	80	18	120		
	yes	0	0	0	0		
Molar relationship Right Class I	no	14	49	12	75	0.199	0.905
	yes	8	31	6	45		
Molar relationship Right Class II	no	8	42	6	56	3.318	0.190
	yes	14	38	12	64		
Molar relationship Right Class III	no	22	69	18	109	6.055	0.048
	yes	0	11	0	11		
Molar relationship Left Class I	no	14	42	11	67	1.107	0.575
	yes	8	38	7	53		
Molar relationship Left Class II	no	8	43	7	58	2.845	0.241
	yes	14	37	11	62		
Molar relationship Left Class III	no	22	75	18	115	2.609	0.271
	yes	0	5	0	5		

**Table 6:** Values of *r* Pearson correlation in our sample. *: *p* < 0.05, statistically significant.

Degree of Scoliosis	Pearson Correlation	*p*-Value
Bilateral cross-bite	−0.192	0.036 *
Cross-bite	0.279	0.002 *
Cross-bite R	0.186	0.042 *
Cross-bite L	0.199	0.029 *
Lower midline deviation	0.165	0.072
Lower midline deviation R	0.23	0.799
Lower midline deviation L	0.189	0.038 *
Contacts on R.C. Bilateral	0.036	0.699
Contacts on R.C. R	0.033	0.717
Contacts on R.C. L		
Molar relationship R CL1	0.015	0.871
Molar relationship R CL2	0.004	0.967
Molar relationship R CL3	0.018	0.842
Molar relationship L CL1	0.022	0.809
Molar relationship L CL2	−0.027	0.77
Molar relationship L CL3	0.012	0.896

**Table 7:** Values of *r* Pearson correlation for cross-bite and midline deviation related to groups of false scoliosis and scoliosis subjects. *: *p* < 0.05, statistically significant.

	False Scoliosis		Scoliosis	
	R conv.	L conv.	R conv.	L conv.
Bilateral cross-bite	0.014	−0.014		
*p*-value	0.9	0.9		
Cross-bite	0.089	−0.089	0.471	−0.0471
*p*-value	0.433	0.433	0.048	0.048
Cross-bite R	0.019	−0.019	0	0
*p*-value	0.868	0.868	1	1
Cross-bite L	0.126	−0.126	0.632	−0.632
*p*-value	0.264	0.264	0.005 *	0.005 *
Midline	0.376	−0.376	0.471	−0.471
*p*-value	0.001 *	0.001 *	0.048	0.048
Midline deviation R	0.148	−0.0148	0.351	−0.351
*p*-value	0.189	0.189	0.153	0.153
Midline deviation L	0.327	−0.0327	0.189	−0.189
*p*-value	0.003 *	0.003 *	0.453	0.453

**Table 8:** Relations between differences in transversal planes and midline deviation in our sample.

Rachis		Lower Midline Deviation		
		R	L	Total
Symmetry	9M	2M		4
16	7F	2F		25%
asymmetric	R conv.			
scapula-humeral	2M			
track	8F	3F	2F	
17	L conv.			
	2M	3M		11
	5F		3F	65%
deflection from	R conv.			
overhanging	5F	2F	1F	
	L conv.			6
13	8F	1F	2F	46%
more than one	R conv.			
transversal plan	5M			
34	14F	8F		
	L conv.			
	5M		2M	10
	10F			29%

**Table 9:** Relations between cross-bites and midline deviations in our study. *: *p* < 0.05, statistically significant.

	Midline Deviation R	Midline Deviation L
Cross-bite R	0.294	0.002
*p*-value	0.001 *	0.986
Cross-bite L	0.078	0.511
*p*-value	0.4	0 *

## References

[1] Grassi F., Pazzaglia U., Pilato G., Zatti G. (2012). Manuale di Ortopedia e Traumatologia.

[2] Fitzgerald R., Kaufer H., Malkani A.L. (2004). Trattato di Ortopedia e Traumatologia.

[3] Trobisch P., Suess O., Schwab F. (2010). Idiopathic scoliosis. Dtsch Arztebl Int..

[4] Kouwenhoven J.W., Castelein R.M. (2008). The pathogenesis of adolescent idiopathic scoliosis: Review of the literature. Spine (Phila Pa 1976).

[5] Cucuzza M.E., Evola G., Evola F.R. (2018). Adolescent Idiopathic Scoliosis: A Minireview. Biomed. J. Sci. Tech. Res..

[6] Hesse K.L., Artun J., Joondeph D.R., Kennedy D.B. (1997). Changes in condylar postition and occlusion associated with maxillary expansion for correction of functional unilateral posterior crossbite. Am. J. Orthod. Dentofac. Orthop..

[7] Langberg B.J., Arai K., Miner R.M. (2005). Transverse skeletal and dental asymmetry in adults with unilateral lingual posterior crossbite. Am. J. Orthod. Dentofac. Orthop..

[8] O’Byrn B.L., Sadowsky C., Schneider B., BeGole E.A. (1995). An evaluation of mandibular asymmetry in adults with unilateral posterior crossbite. Am. J. Orthod. Dentofac. Orthop..

[9] Pinto A.S., Buschang P.H., Throckmorton G.S., Chen P. (2001). Morphological and positional asymmetries of young children with functional unilateral posterior crossbite. Am. J. Orthod. Dentofac. Orthop..

[10] Pirttiniemi P., Kantomaa T., Lahtela P. (1990). Relationship between craniofacial and condyle path asymmetry in unilateral cross-bite patients. Eur. J. Orthod..

[11] Pirttiniemi P., Raustia A., Kantomaa T., Pyhtinen J. (1991). Relationships of bicondylar position to occlusal asymmetry. Eur. J. Orthod..

[12] Solow B., Siersbaek-Nielsen S. (1992). Cervical and craniocervical posture as predictors of craniofacial growth. Am. J. Orthod. Dentofac. Orthop..

[13] Wong M.L., Sandham A., Ang P.K., Wong D.C., Tan W.C., Huggare J. (2005). Craniofacial morphology, head posture, and nasal respiratory resistance in obstructive sleep apnoea: An inter-ethnic comparison. Eur. J. Orthod..

[14] Schmid W., Mongini F., Felisio A. (1991). A computer-based assessment of structural and displacement asymmetries of the mandible. Am. J. Orthod. Dentofac. Orthop..

[15] Bettany-Saltikov J., Weiss H.R., Chockalingam N., Taranu R., Srinivas S., Hogg J., Whittaker V., Kalyan R.V., Arnell T. (2015). Surgical versus non-surgical interventions in people with adolescent idiopathic scoliosis. Cochrane Database Syst. Rev..

[16] Bridwell K.H. (1999). Surgical treatment of idiopathic adolescent scoliosis. Spine (Phila Pa 1976).

[17] Bunge E.M., Juttmann R.E., de Kleuver M., van Biezen F.C., de Koning H.J., The NESCIO Group (2007). Health-related quality of life in patients with adolescent idiopathic scoliosis after treatment: Short-term effects after brace or surgical treatment. Eur. Spine J..

[18] Goldberg C.J., Moore D.P., Fogarty E.E., Dowling F.E. (2002). The natural history of early onset scoliosis. Stud. Health Technol. Inf..

[19] Ricketts R.M. (1955). Abnormal function of the temporal mandibular joint. Am. J. Orthod..

[20] Ricketts R.M. (1989). Provocations and Perceptions in Cranio-Facial Orthop Edics.

[21] Agostino P., Ugolini A., Signori A., Silvestrini-Biavati A., Harrison J.E., Riley P. (2014). Orthodontic treatment for posterior crossbites. Cochrane Database Syst. Rev..

[22] Angle E.H. (1907). Malocclusion of the Teeth.

[23] Saccucci M., Tettamanti L., Mummolo S., Polimeni A., Festa F., Tecco S. (2011). Scoliosis and dental occlusion: A review of the literature. Scoliosis.

[24] Dunn J., Henrikson N.B., Morrison C.C., Blasi P.R., Nguyen M., Lin J.S. (2018). Screening for Adolescent Idiopathic Scoliosis: Evidence Report and Systematic Review for the US Preventive Services Task Force. J. Am. Med. Assoc. JAMA.

[25] Trovato F.M., Roggio F., Szychlinska M.A., Borzì F., Musumeci G. (2016). Clinical Kinesiology and Posturology Applied to a Group of Italian Students. A Morphological Observational Study. J. Funct. Morphol. Kinesiol..

[26] Riseborough E.J., Wynne-Davies R. (1973). A genetic survey of idiopathic scoliosis in Boston, Massachusetts. J. Bone Jt. Surg. Am..

[27] Korbmacher H., Koch L., Eggers-Stroeder G., Kahl-Nieke B. (2007). Associations between orthopaedic disturbances and unilateral crossbite in children with asymmetry of the upper cervical spine. Eur. J. Orthod..

[28] Lippold C., van den Bos L., Hohoff A., Danesh G., Ehmer U. (2003). Interdisciplinary study of orthopedic and orthodontic findings in pre-school infants. J. Orofac. Orthop..

[29] Hirschfelder U., Hirschfelder H. (1983). Effects of scoliosis on the facial bones. Fortschr. Kieferorthop..

[30] Nerder P.H., Bakke M., Solow B. (1999). The functional shift of the mandible in unilateral posterior crossbite and the adaptation of the temporomandibular joints: A pilot study. Eur. J. Orthod..

[31] Tadej G., Engstrom C., Borrman H., Christiansen E.L. (1989). Mandibular condyle morphology in relation to malocclusions in children. Angle Orthod..

[32] Ricketts R.M. (1953). Laminagraphy in the diagnosis of temporomandibular joint disorders. J. Am. Dent. Assoc..

[33] Poikela A., Kantomaa T., Pirttiniemi P. (1997). Craniofacial growth after a period of unilateral masticatory function in young rabbits. Eur. J. Oral Sci..

[34] Huggare J. (1998). Postural disorders and dentofacial morphology. Acta Odontol. Scand..

[35] Azuma Y., Maehara K., Tokunaga T., Hashimoto M., Ieoka K., Sakagami H. (1999). Systemic effects of the occlusal destruction in guinea pigs. In Vivo.

[36] Sambataro S., Cervino G., Fiorillo L., Cicciu M. (2018). Upper First Premolar Positioning Evaluation for the Stability of the Dental Occlusion: Anatomical Considerations. J. Craniofac. Surg..

[37] Sambataro S., Fastuca R., Oppermann N.J., Lorusso P., Baccetti T., Franchi L., Caprioglio A. (2017). Cephalometric changes in growing patients with increased vertical dimension treated with cervical headgear. J. Orofac. Orthop..

[38] Ricketts R.M. (2000). A statement regarding early treatment. Am. J. Orthod. Dentofac. Orthop..

[39] Ricketts R.M. (1979). Dr. Robert M. Ricketts on early treatment (part 1). J. Clin. Orthod..

[40] Ricketts R.M. (1979). Dr. Robert M. Ricketts on early treatment (part 2). J. Clin. Orthod..

[41] Ricketts R.M. (1979). Dr. Robert M. Ricketts on early treatment. (Part 3). J. Clin. Orthod..

[42] Cervino G., Fiorillo L., Laino L., Herford A.S., Lauritano F., Giudice G.L., Fama F., Santoro R., Troiano G., Iannello G. (2018). Oral Health Impact Profile in Celiac Patients: Analysis of Recent Findings in a Literature Review. Gastroenterol. Res. Pract..

[43] Lombardi T., Bernardello F., Berton F., Porrelli D., Rapani A., Camurri Piloni A., Fiorillo L., Di Lenarda R., Stacchi C. (2018). Efficacy of Alveolar Ridge Preservation after Maxillary Molar Extraction in Reducing Crestal Bone Resorption and Sinus Pneumatization: A Multicenter Prospective Case-Control Study. BioMed Res. Int..

[44] Troiano G., Laino L., Cicciu M., Cervino G., Fiorillo L., D’Amico C., Zhurakivska K., Lo Muzio L. (2018). Comparison of Two Routes of Administration of Dexamethasone to Reduce the Postoperative Sequelae After Third Molar Surgery: A Systematic Review and Meta-Analysis. Open Dent. J..

[45] Cervino G., Cicciù M., Biondi A., Bocchieri S., Herford A.S., Laino L., Fiorillo L. (2019). Antibiotic Prophylaxis on Third Molar Extraction: Systematic Review of Recent Data. Antibiotics.

[46] Cervino G., Fiorillo L., Herford A.S., Romeo U., Bianchi A., Crimi S., D’Amico C., De Stefano R., Troiano G., Santoro R. (2019). Molecular Biomarkers Related to Oral Carcinoma: Clinical Trial Outcome Evaluation in a Literature Review. Dis. Mark..

[47] Laino L., Cicciù M., Fiorillo L., Crimi S., Bianchi A., Amoroso G., Monte I.P., Herford A.S., Cervino G. (2019). Surgical Risk on Patients with Coagulopathies: Guidelines on Hemophiliac Patients for Oro-Maxillofacial Surgery. Int. J. Environ. Res. Public Health.

[48] Crimi S., Fiorillo L., Bianchi A., D’Amico C., Amoroso G., Gorassini F., Mastroieni R., Marino S., Scoglio C., Catalano F. (2019). Herpes Virus, Oral Clinical Signs and QoL: Systematic Review of Recent Data. Viruses.

[49] Fiorillo L. (2019). Chlorhexidine Gel Use in the Oral District: A Systematic Review. Gels.

[50] Fiorillo L., De Stefano R., Cervino G., Crimi S., Bianchi A., Campagna P., Herford A.S., Laino L., Cicciù M. (2019). Oral and Psychological Alterations in Haemophiliac Patients. Biomedicines.

